# A Cohort Study of Serum Bilirubin Levels and Incident Non-Alcoholic Fatty Liver Disease in Middle Aged Korean Workers

**DOI:** 10.1371/journal.pone.0037241

**Published:** 2012-05-15

**Authors:** Yoosoo Chang, Seungho Ryu, Yiyi Zhang, Hee Jung Son, Jang-Young Kim, Juhee Cho, Eliseo Guallar

**Affiliations:** 1 Department of Occupational Medicine, Kangbuk Samsung Hospital and Sungkyunkwan University School of Medicine, Seoul, South Korea; 2 Department of Epidemiology, Johns Hopkins Bloomberg School of Public Health, Baltimore, Maryland, United States of America; 3 Health Screening Center, Kangbuk Samsung Hospital and Sungkyunkwan University School of Medicine, Seoul, South Korea; 4 Department of Medicine and Welch Center for Prevention, Epidemiology, and Clinical Research, Johns Hopkins Medical Institutions, Baltimore, Maryland, United States of America; 5 Department of Internal Medicine, Samsung Medical Center and Sungkyunkwan University School of Medicine, Seoul, South Korea; 6 Department of Cardiology, Wonju College of Medicine, Yonsei University, Wonju, South Korea; 7 Institute of Genomic Cohort, Yonsei University, Wonju, South Korea; 8 Samsung Comprehensive Cancer Center, Samsung Medical Center, Seoul, South Korea; 9 Area of Cardiovascular Epidemiology and Population Genetics, National Center for Cardiovascular Research (CNIC), Madrid, Spain; The Chinese University of Hong Kong, Hong Kong

## Abstract

**Background:**

Serum bilirubin may have potent antioxidant and cytoprotective effects. Serum bilirubin levels are inversely associated with several cardiovascular and metabolic endpoints, but their association with nonalcoholic fatty liver disease (NAFLD) has not been investigated except for a single cross-sectional study in a pediatric population. We assessed the prospective association between serum bilirubin concentrations (total, direct, and indirect) and the risk for NAFLD.

**Methods and Findings:**

We performed a cohort study in 5,900 Korean men, 30 to 59 years of age, with no evidence of liver disease and no major risk factors for liver disease at baseline. Study participants were followed in annual or biennial health examinations between 2002 and 2009. The presence of fatty liver was determined at each visit by ultrasonography. We observed 1,938 incident cases of NAFLD during 28,101.8 person-years of follow-up. Increasing levels of serum direct bilirubin were progressively associated with a decreasing incidence of NAFLD. In age-adjusted models, the hazard ratio for NAFLD comparing the highest to the lowest quartile of serum direct bilirubin levels was 0.61 (95% CI 0.54–0.68). The association persisted after adjusting for multiple metabolic parameters (hazard ratio comparing the highest to the lowest quartile 0.86, 95% CI 0.76–0.98; P trend = 0.039). Neither serum total nor indirect bilirubin levels were significantly associated with the incidence of NAFLD.

**Conclusions:**

In this large prospective study, higher serum direct bilirubin levels were significantly associated with a lower risk of developing NAFLD, even adjusting for a variety of metabolic parameters. Further research is needed to elucidate the mechanisms underlying this association and to establish the role of serum direct bilirubin as a marker for NAFLD risk.

## Introduction

Nonalcoholic fatty liver disease (NAFLD) encompasses a spectrum of liver disorders characterized by steatosis with varying degrees of inflammatory activity, necrosis and fibrosis [1], [2]. NAFLD may progress to cirrhosis or hepatocellular carcinoma and is associated with increased risk of cardiovascular morbidity and mortality [3], [4]. NAFLD is closely related to obesity, dyslipidemia, insulin resistance, and type 2 diabetes, and it is considered a manifestation of the metabolic syndrome [5], [6], [7]. While _ENREF_11NAFLD is the most prevalent form of liver disease [8], [9], its pathophysiology is still not fully understood [10] and there is substantial interest in identifying novel determinants of NAFLD incidence.

Bilirubin is a product of heme catabolism [11] that may have potent antioxidant and cytoprotective properties [11], [12]. A growing body of literature shows that higher bilirubin levels are inversely associated with HOMA-IR and insulin levels [13] and with the prevalence of cardiovascular disease [14], [15], [16], [17], [18] and diabetes [19]. Furthermore, an increased expression of heme oxygenase, the enzyme that catalyzes the breakdown of hemoglobin into bilirubin, is associated with reduced adiposity and improved insulin sensitivity in animal models [20], [21]. Only one cross-sectional study, conducted among obese children, has evaluated the association between serum bilirubin and NAFLD [22]. In this study, serum total bilirubin levels were lower in participants with NAFLD compared to those without NAFLD. No information is available on the association of serum bilirubin levels and NAFLD in adults. The goal of our study was thus to evaluate the prospective association between bilirubin concentrations (total, direct, and indirect) and the risk of NAFLD.

**Table 1:** Baseline characteristics of study participants by quartile of serum direct bilirubin (N = 5,900).

	Overall	Direct bilirubin				*P* value trend
		Quartile 1	Quartile 2	Quartile 3	Quartile 4	
**Number**	5900	2196	1,319	924	1461	
**Direct bilirubin, mg/dL** *	0.44 (0.21)	0.25 (0.07)	0.4 (0)	0.5 (0)	0.73 (0.16)	
**Range**	0–1.7	0–	0.4–	0.5–	0.6–1.7	
**Age, years** *	36.8 (4.9)	37.0 (5.1)	36.9 (4.9)	36.6 (4.8)	36.5 (4.7)	0.004
**BMI, kg/m^2^** *	22.9 (2.4)	23.2 (2.4)	22.9 (2.4)	22.7 (2.4)	22.4 (2.3)	<0.001
**Current smoker, %**	43.4	48.2	42.6	42.9	37.0	<0.001
**Alcohol intake, %** ‡	27.5	27.3	27.1	30.8	26.2	0.95
**Regular exercise, %** §	51.4	49.8	50.6	54.3	52.8	0.03
**Hypertension, %**	12.1	12.3	13.0	13.1	10.6	0.20
**Metabolic syndrome, %**	4.8	6.8	4.6	4.6	2.2	<0.001
**Diabetes mellitus, %**	0.6	0.5	0.7	0.7	0.8	0.26
**Cardiovascular dis., %**	0.1	0.1	0.1	0.2	0.1	0.82
**Malignancy, %**	0.2	0.1	0.3	0.2	0.3	0.44
**Lipid lowering agent, %**	0.4	0.5	0.5	0.3	0.2	0.14
**Hemoglobin, g/dL**	15.0 (0.9)	14.9 (0.9)	15.0 (0.8)	15.1 (0.8)	15.2 (0.8)	<0.001
**Leukocyte, ×10^3^/µL**	5.8 (1.4)	6.0 (1.4)	5.8 (1.4)	5.7 (1.4)	5.5 (1.3)	<0.001
**Systolic BP, mmHg** *	114.1 (12.2)	114.1 (12.2)	114.3 (12.0)	114.3 (12.3)	113.7 (12.3)	0.38
**Diastolic BP, mmHg** *	73.8 (9.7)	73.7 (9.7)	74.3 (9.6)	73.8 (10.0)	73.6 (9.6)	0.62
**Glucose, mg/dL** *	90.2 (11.6)	91.7 (10.4)	90.3 (11.4)	90.0 (10.9)	88.1 (13.4)	<0.001
**Uric acid, mg/dL** *	5.84 (1.07)	5.83 (1.07)	5.85 (1.07)	5.86 (1.05)	5.83 (1.09)	0.95
**Tot. cholesterol, mg/dL** *	194.7 (32.2)	201.3 (32.4)	196.2 (32.5)	191.2 (31.3)	185.7 (29.6)	<0.001
**LDL-C, mg/dL** *	116.3 (27.7)	120.8 (27.7)	117.4 (27.7)	114.0 (27.6)	110.0 (26.5)	<0.001
**HDL-C, mg/dL** *	53.4 (11.6)	51.1 (11.0)	53.2 (11.8)	54.0 (11.1)	56.8 (11.7)	<0.001
**Triglycerides, mg/dL** †	109.0 (82.0–150.0)	122.0 (90.0–166.0)	113.0 (84.0–153.0)	104.0 (80.0–142.0)	96.0 (74.0–128.0)	<0.001
**Total bilirubin, mg/dL**	1.18 (0.48)	0.80 (0.20)	1.07 (0.18)	1.28 (0.21)	1.76 (0.48)	<0.001
**Indirect bilirubin, mg/dL**	0.73 (0.31)	0.55 (0.18)	0.67 (0.18)	0.78 (0.21)	1.03 (0.37)	<0.001
**Albumin, g/dL**	4.43 (0.19)	4.43 (0.19)	4.43 (0.19)	4.43 (0.19)	4.43 (0.19)	0.59
**ALT, IU/L** †	20.0 (16.0–25.0)	21.0 (17.0–27.0)	21.0 (17.0–26.0)	19.0 (16.0–25.0)	19.0 (16.0–23.0)	<0.001
**AST, IU/L** †	21.0 (19.0–24.0)	21.0 (19.0–24.0)	21.0 (19.0–24.0)	21.0 (19.0–24.0)	21.0 (18.0–24.0)	0.01
**GGT, IU/L** †	20.0 (15.0–27.0)	21.0 (16.0–28.0)	20.0 (15.0–28.0)	20.0 (15.0–26.0)	19.0 (15.0–25.0)	<0.001
**ALP, IU/L** †	54.0 (47.0–63.0)	55.0 (47.0–64.0)	54.0 (47.0–63.0)	53.0 (47.0–62.0)	53.0 (46.0–62.0)	<0.001
**hsCRP, mg/L** †	0.40 (0.20–0.80)	0.50 (0.20–1.00)	0.40 (0.20–0.80)	0.40 (0.20–0.70)	0.30 (0.20–0.60)	<0.001
**Insulin, µU/dL** †	6.33 (5.10–8.20)	6.89 (5.36–8.99)	6.41 (5.17–8.20)	6.03 (4.92–7.60)	5.88 (4.83–7.46)	<0.001
**HOMA2-IR** †	0.82 (0.66–1.07)	0.90 (0.70–1.17)	0.83 (0.67–1.07)	0.78 (0.64–1.00)	0.76 (0.63–0.96)	<0.001

Data are.

* means (standard deviation),

† medians (interquartile range), or percentages.

Abbreviations: ALT, alanine aminotransferase; AST, aspartate aminotransferase; BMI, body mass index; BP, blood pressure; GGT, gamma-glutamyltranspeptidase; ALP, alkaline phosphatase; HDL-C, high-density lipoprotein-cholesterol; hsCRP, high sensitivity C-reactive protein; HOMA2-IR, homeostasis model assessment 2 of insulin resistance; LDL-C: low-density lipoprotein-cholesterol.

‡ ≥20 g of ethanol per day.

§ ≥1 time/week.

## Methods

### Study Population

The study population consisted of male workers from one of the largest semiconductor manufacturing companies in Korea and its 13 affiliates [23], [24]. In Korea, the Industrial Safety and Health Law requires that employees participate in annual or biennial health examinations. Study participants were all male workers 30 to 59 years of age from the above mentioned semiconductor companies who participated in a comprehensive health examination at the Kangbuk Samsung Hospital in Seoul, Korea in 2002 (N = 15,347). For this analysis, we excluded 5053 participants with ultrasonographic evidence of fatty liver at baseline, 585 participants with missing data on bilirubin or other covariates at baseline, and 1611 participants not attending any follow-up visit after the baseline visit. The sample size for the eligible population was 8,871.

This study was approved by the Institutional Review Board of the Kangbuk Samsung Hospital. The informed consent requirement was exempted by the Institutional Review Board because researchers only accessed retrospectively a de-identified database for analysis purposes.

**Figure 1:**
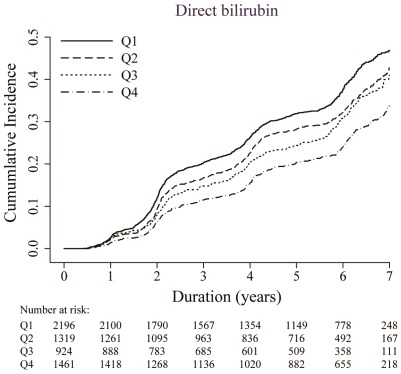
Cumulative incidence of non-alcoholic fatty liver disease by quartile of serum direct bilirubin concentration.

**Figure 2:**
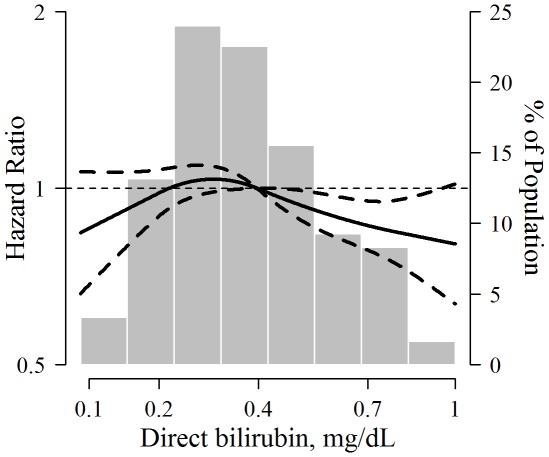
Multivariable-adjusted hazard ratio (95% confidence interval) for non-alcoholic fatty liver disease by concentration of serum direct bilirubin. Risk trends were estimated using restricted quadratic splines with knots at the 5^th^, 50^th^, and 95^th^ percentiles of the direct bilirubin distribution (the reference value with hazard ratio = 1 was set at the 50^th^ percentile). The horizontal dotted line indicates a hazard ratio of 1. The spline regression model was adjusted for age, BMI, smoking, alcohol intake, exercise, HDL cholesterol, triglycerides, glucose, insulin, and uric acid. The histogram represents the frequency distribution of direct bilirubin concentrations in the study population.

**Table 2:** Hazard ratios (95% confidence intervals) for incident non-alcoholic fatty liver disease by serum bilirubin quartiles (N = 5,900).

	Person-years	No. of incident cases	Age-adjusted HR (95% CI)	Multivariate HR^*^ (95% CI)	
				Model 1	Model 2
**Direct bilirubin (mg/dL)**					
** 0–**	10127.5	841	1.00 (reference)	1.00 (reference)	1.00 (reference)
** 0.4–**	6220.3	433	0.83 (0.73–0.93)	0.87 (0.77–0.98)	0.94 (0.83–1.05)
** 0.5–**	4413.7	286	0.77 (0.67–0.88)	0.86 (0.75–0.98)	0.97 (0.85–1.12)
** 0.6–1.7**	7340.4	378	0.61 (0.54–0.68)	0.73 (0.65–0.83)	0.86 (0.76–0.98)
** P for trend**			<0.001	<0.001	0.039
**Indirect bilirubin (mg/dL)**					
** 0–**	7729.9	565	1.00 (reference)	1.00 (reference)	1.00 (reference)
** 0.6–**	8971.7	658	1.00 (0.89–1.12)	0.99 (0.89–1.11)	1.06 (0.95–1.19)
** 0.7–**	5929.4	377	0.87 (0.76–0.99)	0.92 (0.81–1.05)	0.99 (0.87–1.13)
** 0.9–3.5**	5470.8	338	0.84 (0.74–0.97)	0.91 (0. 79–1.04)	0.99 (0.86–1.14)
** P for trend**			0.003	0.10	0.72
**Total bilirubin (mg/dL)**					
** 0.3–**	9607.7	749	1.00 (reference)	1.00 (reference)	1.00 (reference)
** 1.0–**	5713.5	431	0.96 (0.85–1.08)	0.97 (0.86–1.09)	1.04 (0.92–1.17)
** 1.2–**	6531.6	388	0.75 (0.67–0.85)	0.82 (0.72–0.93)	0.91 (0.80–1.03)
** 1.5–4.4**	6249.0	370	0.76 (0.67–0.86)	0.87 (0.76–0.98)	0.97 (0.85–1.10)
** P for trend**			<0.001	0.002	0.31

Model 1: Adjusted for age, BMI, current smoking, alcohol intake, exercise, diabetes mellitus, history of cardiovascular disease and history of malignancy.

Model 2: Further adjusted for HDL cholesterol, triglycerides, glucose, insulin, and uric acid.

Abbreviations: BMI, body mass index; CI, confidence intervals; HDL-C, high-density lipoprotein-cholesterol; HR, hazard ratios.

### Measurements

Baseline and follow-up examinations were conducted at the Kangbuk Samsung Hospital Health Screening Center. Study participants were examined annually or biennially until December 2009. Baseline and follow-up health examinations collected data on medical history, medication use, health-related behaviors, physical measurements, and serum biochemical measurements [23]. Questions pertaining to alcohol intake included weekly frequency of alcohol consumption and usual daily amount of consumption. Questionnaire data were also used to identify current smokers and to assess the weekly frequency of moderate- or vigorous-intensity physical activity. Body weight was measured with light clothing and without shoes to the nearest 0.1 kilogram using a digital scale. Height was measured to the nearest 0.1 centimeter. Body mass index was calculated as weight in kilograms divided by height in meters squared. Trained nurses measured sitting blood pressure with a mercury sphygmomanometer.

Blood specimens were sampled from the antecubital vein after more than 12 hours of fasting. Serum levels of glucose, uric acid, total cholesterol, triglycerides, low-density lipoprotein (LDL) cholesterol, high-density lipoprotein (HDL) cholesterol, gamma-glutamyltransferase (GGT), alanine aminotransferase (ALT), aspartate aminotransferase (AST), alkaline phosphatase and total bilirubin and direct bilirubin were measured using Bayer Reagent Packs on an automated chemistry analyzer (Advia 1650™ Autoanalyzer; Bayer Diagnostics, Leverkusen, Germany). Serum total and direct bilirubin were measured with the vanadate oxidation method. The total coefficients of variation for total bilirubin determinations were <2.1% and <1.6% for low-level and high-level quality control samples during the study period, respectively. The total coefficients of variation for direct bilirubin determinations were <5.9% and <3.3% for low-level and high-level quality control samples, respectively. Insulin was measured using an immunoradiometric assay (Biosource, Nivelles, Belgium). Insulin resistance was assessed with the homeostasis model assessment 2 of insulin resistance (HOMA2-IR) [26]. High sensitivity-C reactive protein (hsCRP) was analyzed by particle-enhanced immunonephelometry with the BNII™ System (Dade Behring, Marburg, Germany) using a lower detection limit of 0.175 mg/L. The clinical laboratory has been accredited and participates annually in inspections and surveys by the Korean Association of Quality Assurance for Clinical Laboratories.

Abdominal ultrasounds were performed with a 3.5-MHz transducer (Logic Q700 MR, GE, Milwaukee, WI) by 12 experienced radiologists who were unaware of the aims of the study and blinded to laboratory values. Images were captured in a standard fashion with the patient in the supine position with the right arm raised above the head [24]. An ultrasonographic diagnosis of fatty liver (USFL) was defined as the presence of a diffuse increase of fine echoes in the liver parenchyma compared with the kidney or spleen parenchyma [27]. Diagnoses were made by the radiologists using liver images. The study outcome was the development of NAFLD, defined as the presence of new cases of USFL appearing in this cohort free of liver disease or traditional risk factors for liver disease at baseline.

Metabolic syndrome was defined according to National Cholesterol Education Program Adult Treatment Panel III [28] as the presence of three or more of the following five abnormalities: 1) abdominal obesity; 2) fasting blood glucose ≥110 mg/dL; 3) fasting serum triglycerides ≥150 mg/dL (to convert to mmol/L, multiply by 0.01129); 4) HDL cholesterol <40 mg/dL (to convert to mmol/L, multiply by 0.02586); and 5) blood pressure ≥130/85 mm Hg. Because waist circumference measurements were not available for all participants, we substituted abdominal obesity with overall adiposity as a BMI of ≥25 kg/m^2^, which was proposed as a cut-off for diagnosis of obesity in Asian populations [29]. Diabetes mellitus was defined by a self-reported physician diagnosis, self-reported medical therapy for diabetes mellitus or fasting serum blood glucose greater than 126 mg/dL. Cardiovascular disease (any type of heart disease or stroke) and malignancy was defined by a self-reported physician diagnosis. Hypertension was defined as use of antihypertensive medication, self-reported history or blood pressure ≥140/90 mm Hg.

### Statistical Analyses

Since NAFLD is a diagnosis of exclusion, the primary analyses were based on the subset of study participants who did not have any evidence of liver disease or any risk factors for liver disease at baseline. For the primary analyses, we further excluded 9 participants taking medications for hepatitis; 457 participants with positive serologic markers for hepatitis B or C virus; 140 participants with ultrasonographic findings of chronic liver disease, liver cirrhosis, cholelithiasis, or abnormal dilatation of the biliary tree; 6 participants who, in the past year, had taken medications that may induce NAFLD (e.g., steroids, immune suppressants, anticonvulsants, and tetracyclines); 1473 participants reporting an alcohol intake ≥20 g/day [2], [25]; 1082 participants with elevated alanine aminotransferase (ALT) levels (>35 U/L); and 271 participants with elevated aspartate aminotransferase (AST) levels (>40 U/L). Because some individuals met more than one exclusion criterion, the total number of men eligible for the study at baseline was 5900. As sensitivity analyses, we also performed analyses in the overall study population (N = 8,871), adjusted for the presence of liver disease or of risk factors for liver disease at baseline.

One-way ANOVA and χ^2^-tests were used to compare the characteristics of study participants at baseline by quartiles of serum direct (<0.4, 0.4–<0.5, 0.5–<0.6, and ≥0.6 mg/dL), indirect (<0.6, 0.6–<0.7, 0.7–<0.9, ≥0.9 mg/dL), or total bilirubin (<1.0, 1.0–<1.2, 1.2–<1.5, and ≥1.5 mg/dL). The distribution of continuous variables was evaluated and appropriate transformations were done in the analysis as needed. Follow-up extended from the baseline exam until the development of NAFLD or the last health exam conducted for each participant. The average follow-up period for participants who did not develop NAFLD was 5.4 years. The cumulative incidence of NAFLD by quartile of total, indirect and direct bilirubin concentrations was assessed by Kaplan-Meier plots. We then estimated adjusted hazard ratios (and 95% confidence intervals) for incident NAFLD comparing the three highest quartiles of baseline serum bilirubin to the lowest quartile using proportional hazards regression. Since we knew that NAFLD had occurred between two visits but did not know the precise time of NAFLD development, we used a parametric Cox model to take into account this type of interval censoring [30]. In these models, the baseline hazard function was parameterized with restricted cubic splines in log time with four degrees of freedom.

Proportional hazards models were initially adjusted for age, then for body mass index, smoking, alcohol intake, exercise, diabetes mellitus, history of cardiovascular disease, history of malignancy, and then for other metabolic markers including fasting blood glucose, systolic blood pressure, triglycerides, HDL-cholesterol, HOMA-IR and C reactive protein. For testing linear risk trends, we used the quartile rank as a continuous variable in the regression models. We checked the proportional hazards assumption by examining graphs of estimated log(-log) survival. In addition, we estimated the hazard ratio for incident NAFLD associated with an increase in 1 mg/dL of serum bilirubin modeled as a continuous variable. We further explored the shape of the dose-response relation between serum bilirubin concentration and NAFLD incidence by using restricted quadratic splines with knots at the 5^th^, 50^th^, and 95^th^ percentiles of the bilirubin distribution. Statistical analyses were performed using Stata version 11 (StataCorp LP, College Station, TX, USA). All P values reported were 2-tailed, and statistical significance was set at P<0.05.

## Results

At baseline, the mean (SD) age, body mass index, and serum total, direct and indirect bilirubin levels of study participants were 36.8 (4.9) years, 22.9 (2.4) kg/m^2^, 1.2 (0.5) mg/dL, 0.4 (0.2) mg/dL, and 0.7 (0.3) mg/dL, respectively (Table 1). The prevalences of current smoking, hypertension and metabolic syndrome were 43.4, 12.1 and 4.8%, respectively. Serum direct bilirubin levels were inversely associated with a variety of metabolic parameters, including body mass index, glucose, total cholesterol, LDL-cholesterol, triglycerides, liver enzymes, hsCRP, insulin and HOMA-IR, and positively associated with HDL-cholesterol levels (Table 1). Serum direct bilirubin levels were also inversely associated with current smoking and with the prevalence of the metabolic syndrome. Serum total and indirect bilirubin levels showed a similar association pattern with demographic, anthrompometric, and cardiometabolic parameters compared to serum direct bilirubin (Tables S1 and S2), but serum total and indirect bilirubin levels were positively associated with uric acid while serum direct bilirubin levels were not. Serum indirect bilirubin levels were positively associated with blood pressure while total and direct bilirubin levels were not.

During 28,101.8 person-years of follow-up, 1,938 participants developed NAFLD (rate 6.9 per 100 person-years). Decreasing levels of serum direct bilirubin were progressively associated with increasing incidence of NAFLD (Figure 1 and Table 2). In age-adjusted models, the hazard ratios for NAFLD comparing quartiles 2–4 vs. quartile 1 of serum direct bilirubin were 0.83 (95% confidence interval 0.73–0.93), 0.77 (0.67–0.88) and 0.61 (0.54–0.68), respectively. After adjusting for body mass index, smoking, alcohol intake, exercise, diabetes mellitus, history of cardiovascular diseae and malignancy, the hazard ratio for NAFLD in the highest quartile compared to the lowest was 0.73 (0.65–0. 83; P trend<0.001). The association also persisted after adjusting for multiple metabolic parameters (hazard ratio comparing the highest to the lowest quartile 0.86, 95% CI 0.76–0.98; P trend = 0.039). Similar associations were also observed when the analysis was restricted to participants without metabolic syndrome, insulin resistance (HOMA2-IR≥1.07, highest quartile in the study population) or obesity (BMI≥25 kg/m^2^) at baseline (data not shown). When serum direct bilirubin was introduced as a continuous variable in multivariate models, the hazard ratio for NAFLD associated with an increase of 1 mg/dL in direct bilirubin was 0.77 (95% CI 0.61–0.98; P = 0.037). Spline regression analyses suggested that the association of bilirubin with incident NAFLD was nonlinear (P-value for non-linear spline terms = 0.04), but an inverse association between bilirubin and NAFLD was still evident above direct bilirubin concentrations of 0.30 mg/dL, representing the 25^th^ percentile of the direct bilirubin distribution (Figure 2).

In both the age-adjusted model and the model further adjusted for body mass index, smoking, alcohol intake and exercise, diabetes mellitus, history of cardiovascular diseae and malignancy, serum total bilirubin levels were inversely associated with a significantly increased risk for NAFLD (Table 2). This association, however, did not persist after adjusting for multiple metabolic parameters. Serum levels of indirect bilirubin were not associated with incident NAFLD after adjusting for age, BMI, smoking, alcohol intake, exercise, diabetes mellitus, history of cardiovascular disease and malignancy (Table 2). After exclusion of participants (n = 134) with elevated GGT of ≥63 IU/L or elevated alkaline phosphatase of ≥130 IU/L (upper reference intervals used at Kangbuk Samsung Hospital, respectively), the hazard ratio for NAFLD comparing the highest to the lowest quartile of serum direct bilirubin levels didn't change qualitatively (0.84, 95% CI 0.75–0.97). In sensitivity analyses, we repeated the analyses after including all eligible participants that had liver disease or risk factors for liver disease at baseline except for NAFLD, with consistent findings (Tables S3 and S4).

## Discussion

In this longitudinal study of Korean healthy men, higher serum direct bilirubin levels were significantly associated with a lower risk of developing new cases of NAFLD even after adjusting for obesity, insulin resistance, and other metabolic parameters. Neither total nor indirect bilirubin levels were significantly associated with NAFLD incidence. Our study, the first one to show an association between serum direct bilirubin and the development of NAFLD, suggests that prediagnostic levels of serum bilirubin may play a role in progression to NAFLD and further contributes to an accumulating body of evidence showing that increased serum bilirubin levels are inversely associated with health outcomes related to oxidative stress including coronary artery disease, peripheral vascular disease and cancer [31], [32], [33].

Bilirubin has long been considered simply as the metabolic end product of heme catabolism, but it is becoming increasingly evident that bilirubin can act as a potent antioxidant under physiologic conditions [11] and may play an important role in protecting from oxidative stress [12], [34]. In our study, current smoking was inversely associated with all types of serum bilirubin concentrations. Cigarette smoke might enhance bilirubin oxidation, resulting in decreased bilirubin concentration [35]. Oxidative stress seems to be a major contributor to the pathogenesis of NAFLD [36], even in normal glucose tolerance subjects without insulin resistance [37]. Indeed, increases in reactive oxygen species levels may precede the onset of insulin resistance [38] and lead to diminished insulin action [39], and lipid peroxidation, inflammation and cellular injury [40]. In addition to oxidative stress, NAFLD is also related to insulin resistance. While low serum bilirubin levels are also associated with increased HOMA-IR and insulin levels [13], the association between increased direct bilirubin and lower incidence of NAFLD in our study persisted after adjusting for a variety of measures of insulin resistance and after restricting the analysis to participants with no evidence of insulin resistance, suggesting that the protective role of direct bilirubin is independent of insulin resistance.

Bilirubin may also be linked to NAFLD via fatty acid metabolism. Elevated de novo lipogenesis and peripheral fatty acids mainly derived from lipolysis of adipose tissue contribute to the accumulation of hepatic fat in NAFLD [3], [41]. Bilirubin showed antilipolytic activity in animal models [42], [43] probably due to direct inhibition of triglyceride lipase [43]. On the other hand, serum bilirubin concentrations can influence free fatty acid metabolism [44]. Furthermore, free fatty acids may interfere with bilirubin metabolism at any stage, from blood transport to its conjugation by the hepatocyte [45], [46], and the control of serum bilirubin concentrations may be linked to lipid metabolism [45].

Total bilirubin consists of direct (conjugated) and indirect (unconjugated) bilirubin. Unconjugated bilirubin is derived from reduction of biliverdin, a product of heme degradation, by biliverdin reductase [47]. Unconjugated bilirubin is strongly bound to serum albumin, which transports it to the liver. In hepatocytes, bilirubin is conjugated with one or two molecules of glucuronic acid, a reaction catalyzed by bilirubin-UDP-glucuronosyltransferase [48]. In comparion to unconjugated bilirubin, conjugated bilirubin is soluble in serum and only weakly bound to albumin, and thus more easily available in active form compared to indirect bilirubin [49]. While our study found that the inverse association between bilirubin and incident NAFLD was restricted to direct bilirubin, most previous studies of bilirubin levels and cardiometabolic endpoints measured only total bilirubin without separation of bilirubin types. There is only limited data showing a differentially protective effect of direct bilirubin on metabolic syndrome, but fasting direct bilirubin levels were also inversely associated with the metabolic syndrome [50] and with their components [51] in Korean men and women.

There are several limitations to our study. First, we used USFL in participants without other apparent causes of fatty liver disease as our marker of NAFLD without histologic confirmation for fatty liver. Although USFL seems to be a reliable marker of biopsy-detected fatty liver [27], [52], measurement error in outcome assessment in our study may have diluted the observed associations. Second, total and direct bilirubin were measured a single time, thus adding further measurement error and potential attenuation of the associations. Third, alcohol intake, a common cause of fatty liver in Korean men, was self-reported and was likely underestimated. However, the relationship between serum direct bilirubin and incident NAFLD was similar after excluding participants with serum GGT of ≥40 U/I (75^th^ percentile of GGT in our study population [23]), a commonly used marker of alcohol consumption. _ENREF_24 Finally, we studied young, healthy working Korean men, a population with only moderate prevalence of obesity and insulin resistance but relatively high prevalence of NAFLD and our findings may not be generalizable to women or to men in other populations.

Major strengths of our study include the large sample size, its prospective design, and the availability of detailed health exam and laboratory information, that allowed us to identify a cohort of participants with very low probability of traditional liver risk factors and of liver disease at baseline and to adjust for a variety of cardiometabolic risk factors. Our understanding of bilirubin function is expanding rapidly, and several epidemiological studies have reported potentially protective effects elevated bilirubin concentrations on diabetes, cardiovascular disease or cancer [14], [15], [16], [17], [18], [19], [53]. Our study extends the range of health outcomes inversely associated with bilirubin concentrations and avoids the limitations of cross-sectional studies.

In conclusion, we found an inverse association between serum direct bilirubin and the incidence of NAFLD in a cohort of young, healthy Korean males. The cohort design and the sensitivity analysis restricting the sample to non-obese patients with no evidence of insulin resistance further suggest that this association is independent of other established causes of NAFLD. Our findings thus suggest that serum direct bilirubin levels may play a role in preventing NAFLD, but further research is needed to understand potential protective mechanisms of bilirubin on NAFLD and other chronic diseases.

## Supporting Information

Table S1 **Baseline characteristics of study participants by quartile of serum total bilirubin (N = 5,900).**
(DOC)

Table S2 **Baseline characteristics of study participants by quartile of serum indirect bilirubin (N = 5,900).**
(DOC)

Table S3 **Baseline characteristics of study participants by quartile of serum direct bilirubin (N = 8,871).**
(DOC)

Table S4 **Hazard ratios (95% confidence intervals) for incident non-alcoholic fatty liver disease by serum bilirubin quartiles (N = 8,871).**
(DOC)
