# Supplementary material for: A Cohort Study of Serum Bilirubin Levels and Incident Non-Alcoholic Fatty Liver Disease in Middle Aged Korean Workers
Source: PLoS One. 2012 May 15;7(5):e37241. doi: 10.1371/journal.pone.0037241 (PMC3352875; doi:10.1371/journal.pone.0037241)
Supplement: Table S1 — Baseline characteristics of study participants by quartile of serum total bilirubin (N = 5,900). (DOC) [file pone.0037241.s001.doc]

**Table S1. Baseline characteristics of study participants by quartile of serum total bilirubin (N = 5,900).**

|  | Overall | Total bilirubin | | | | | *P* value for trend |
| --- | --- | --- | --- | --- | --- | --- | --- |
|  |  | Quartile 1 | | Quartile 2 | Quartile 3 | Quartile 4 |  |
| Number | 5900 | | 2065 | 1226 | 1335 | 1274 |  |
| Total bilirubin, mg/dL^*^ | 1.18 (0.48) | | 0.75 (0.14) | 1.05 (0.05) | 1.29 (0.08) | 1.88 (0.43) |  |
| Range | 0.3 – 4.4 | | 0.3 – | 1.0 – | 1.2 – | 1.5 – 4.4 |  |
| Age, years^*^ | 36.8 (4.9) | | 37.0 (5.0) | 37.1 (5.0) | 36.7 (4.7) | 36.3 (4.7) | <0.001 |
| BMI, kg/m^2*^ | 22.9 (2.4) | | 23.1 (2.4) | 23.0 (2.5) | 22.7 (2.4) | 22.5 (2.3) | <0.001 |
| Current smoker, % | 43.4 | | 50.0 | 42.9 | 39.5 | 37.1 | <0.001 |
| Alcohol intake, %^‡^ | 27.5 | | 27.1 | 27.8 | 31.0 | 24.3 | 0.50 |
| Regular exercise, %^§^ | 51.4 | | 51.5 | 50.3 | 51.5 | 52.1 | 0.70 |
| Hypertension, % | 12.1 | | 11.9 | 12.5 | 12.4 | 11.9 | 0.96 |
| Metabolic syndrome, % | 4.8 | | 6.2 | 4.9 | 4.4 | 2.9 | <0.001 |
| Diabetes mellitus, % | 0.6 | | 0.4 | 0.9 | 0.6 | 0.8 | 0.31 |
| Cardiovascular disease, % | 0.1 | | 0.2 | 0.1 | 0.2 | 0.2 | 0.87 |
| Malignancy, % | 0.2 | | 0.3 | 0.1 | 0.2 | 0.2 | 0.81 |
| Lipid lowering agent, % | 0.4 | | 0.4 | 0.5 | 0.5 | 0.2 | 0.44 |
| Hemoglobin, g/dL | 15.0 (0.9) | | 14.9 (0.9) | 15.0 (0.8) | 15.1 (0.8) | 15.3 (0.8) | <0.001 |
| Leukocyte, x10^3^/μL | 5.8 (1.4) | | 6.0 (1.4) | 5.8 (1.5) | 5.6 (1.3) | 5.7 (1.3) | <0.001 |
| Systolic BP, mmHg^*^ | 114.1 (12.2) | | 113.7 (11.9) | 114.5 (12.3) | 114.0 (12.2) | 114.3 (12.5) | 0.27 |
| Diastolic BP, mmHg^*^ | 73.8 (9.7) | | 73.5 (9.5) | 74.3 (9.8) | 73.7 (9.9) | 74.1 (9.5) | 0.21 |
| Glucose, mg/dL^*^ | 90.2 (11.6) | | 91.3 (10.0) | 90.5 (12.9) | 89.7 (9.9) | 88.7 (13.8) | <0.001 |
| Uric acid, mg/dL^*^ | 5.84 (1.07) | | 5.79 (1.07) | 5.82 (1.10) | 5.86 (1.03) | 5.91 (1.09) | 0.001 |
| Total cholesterol, mg/dL^*^ | 194.7 (32.2) | | 196.8 (32.1) | 195.7 (33.0) | 193.7 (32.4) | 191.4 (30.9) | <0.001 |
| LDL-C, mg/dL^*^ | 116.3 (27.7) | | 117.4 (27.5) | 117.0 (28.3) | 115.8 (28.0) | 114.2 (27.1) | 0.001 |
| HDL-C, mg/dL^*^ | 53.4 (11.6) | | 52.1 (11.6) | 53.1 (11.6) | 53.9 (11.1) | 55.5 (11.6) | <0.001 |
| Triglycerides, mg/dL^†^ | 109.0 (82.0-150.0) | | 116.0 (86.0-161.0) | 113.0 (85.0-155.0) | 104.0 (79.0-142.0) | 103.0 (78.0-137.0) | <0.001 |
| Direct bilirubin, mg/dL | 0.44 (0.21) | | 0.27 (0.09) | 0.39 (0.09) | 0.49 (0.10) | 0.72 (0.19) | <0.001 |
| Indirect bilirubin, mg/dL | 0.73 (0.31) | | 0.48 (0.11) | 0.66 (0.09) | 0.80 (0.11) | 1.16 (0.32) | <0.001 |
| Albumin, g/dL | 4.43 (0.19) | | 4.41 (0.19) | 4.43 (0.19) | 4.44 (0.19) | 4.45 (0.20) | <0.001 |
| ALT, IU/L^†^ | 20.0 (16.0-25.0) | | 22.0 (16.0-26.0) | 21.0 (16.0-26.0) | 20.0 (16.0-25.0) | 20.0 (16.0-24.0) | <0.001 |
| AST, IU/L^†^ | 21.0 (19.0-24.0) | | 21.0 (19.0-24.0) | 21.0 (19.0-24.0) | 21.0 (19.0-24.0) | 21.0 (19.0-24.0) | 0.74 |
| GGT, IU/L^†^ | 20.0 (15.0-27.0) | | 20.0 (15.0-28.0) | 20.0 (15.0-28.0) | 20.0 (15.0-26.0) | 19.0 (15.0-25.0) | <0.001 |
| ALP, IU/L† | 54.0 (47.0-63.0) | | 55.0 (48.0-64.0) | 54.0 (47.0-63.0) | 54.0 (46.0-62.0) | 54.0 (46.0-62.0) | <0.001 |
| hsCRP, mg/L^†^ | 0.40 (0.20-0.80) | | 0.50 (0.20-1.00) | 0.40 (0.20-0.80) | 0.40 (0.20-0.70) | 0.30 (0.20-0.60) | <0.001 |
| Insulin, μU/dL | 6.33 (5.10-8.20) | | 6.82 (5.36-8.93) | 6.39 (5.12-8.36) | 5.97 (4.93-7.60) | 6.00 (4.87-7.65) | <0.001 |
| HOMA2-IR† | 0.82 (0.66-1.07) | | 0.89 (0.71-1.17) | 0.83 (0.67-1.08) | 0.78 (0.64-0.99) | 0.77 (0.63-0.99) | <0.001 |

Data are ^*^means (standard deviation), ^†^medians (interquartile range), or percentages.

Abbreviations: ALT, alanine aminotransferase; AST, aspartate aminotransferase; BMI, body mass index; BP, blood pressure; GGT, gamma-glutamyltranspeptidase; HDL-C, high-density lipoprotein-cholesterol; hsCRP, high sensitivity C-reactive protein; HOMA-IR, homeostasis model assessment of insulin resistance; LDL-C: low-density lipoprotein-cholesterol.

^‡^ ≥20 g of ethanol per day.

^§^ ≥1 time/week.
