# Supplementary material for: A Cohort Study of Serum Bilirubin Levels and Incident Non-Alcoholic Fatty Liver Disease in Middle Aged Korean Workers
Source: PLoS One. 2012 May 15;7(5):e37241. doi: 10.1371/journal.pone.0037241 (PMC3352875; doi:10.1371/journal.pone.0037241)
Supplement: Table S2 — Baseline characteristics of study participants by quartile of serum indirect bilirubin (N = 5,900). (DOC) [file pone.0037241.s002.doc]

**Table S2. Baseline characteristics of study participants by quartile of serum indirect bilirubin (N = 5,900).**

|  | Overall | Indirect bilirubin | | | | | *P* value for trend |
| --- | --- | --- | --- | --- | --- | --- | --- |
|  |  | Quartile 1 | | Quartile 2 | Quartile 3 | Quartile 4 |  |
| Number | 5900 | | 1642 | 1,921 | 1,207 | 1,130 |  |
| Indirect bilirubin, mg/dL^*^ | 0.73 (0.31) | | 0.43 (0.08) | 0.65 (0.05) | 0.84 (0.05) | 1.21 (0.31) |  |
| Range | 0 – 3.5 | | 0 – | 0.6 – | 0.7 – | 0.9 – 3.5 |  |
| Age, years^*^ | 36.8 (4.9) | | 37.0 (4.9) | 37.0 (5.0) | 36.6 (4.8) | 36.3 (4.7) | <0.001 |
| BMI, kg/m^2*^ | 22.9 (2.4) | | 23.0 (2.4) | 23.0 (2.4) | 22.8 (2.4) | 22.6 (2.3) | <0.001 |
| Current smoker, % | 43.4 | | 51.2 | 42.9 | 39.5 | 36.8 | <0.001 |
| Alcohol intake, %^‡^ | 27.5 | | 27.3 | 27.9 | 30.2 | 24.3 | 0.35 |
| Regular exercise, %^§^ | 51.4 | | 52.7 | 51.3 | 50.5 | 50.6 | 0.22 |
| Hypertension, % | 12.1 | | 11.5 | 12.1 | 13.3 | 12.0 | 0.42 |
| Metabolic syndrome, % | 4.8 | | 5.5 | 4.9 | 4.9 | 3.5 | 0.02 |
| Diabetes mellitus, % | 0.6 | | 0.7 | 0.5 | 0.6 | 0.9 | 0.52 |
| Cardiovascular disease, % | 0.1 | | 0.2 | 0.1 | 0.2 | 0.1 | 0.63 |
| Malignancy, % | 0.2 | | 0.4 | 0.2 | 0.0 | 0.3 | 0.30 |
| Lipid lowering agent, % | 0.4 | | 0.3 | 0.5 | 0.4 | 0.4 | 0.62 |
| Hemoglobin, g/dL | 15.0 (0.9) | | 14.8 (0.9) | 15.0 (0.8) | 15.2 (0.8) | 15.3 (0.8) | <0.001 |
| Leukocyte, x10^3^/μL | 5.8 (1.4) | | 6.0 (1.5) | 5.8 (1.4) | 5.6 (1.3) | 5.7 (1.3) | <0.001 |
| Systolic BP, mmHg^*^ | 114.1 (12.2) | | 113.5 (11.6) | 114.2 (12.4) | 114.4 (12.4) | 114.5 (12.4) | 0.02 |
| Diastolic BP, mmHg^*^ | 73.8 (9.7) | | 73.3 (9.4) | 74.0 (9.8) | 73.9 (9.9) | 74.2 (9.5) | 0.03 |
| Glucose, mg/dL^*^ | 90.2 (11.6) | | 91.0 (11.3) | 90.3 (10.5) | 90.0 (10.8) | 89.1 (14.2) | <0.001 |
| Uric acid, mg/dL^*^ | 5.84 (1.07) | | 5.76 (1.08) | 5.81 (1.06) | 5.89 (1.04) | 5.95 (1.09) | <0.001 |
| Total cholesterol, mg/dL^*^ | 194.7 (32.2) | | 191.6 (31.4) | 196.1 (33.0) | 196.9 (32.3) | 194.4 (31.4) | 0.007 |
| LDL-C, mg/dL^*^ | 116.3 (27.7) | | 113.4 (27.1) | 117.4 (28.3) | 118.0 (27.7) | 116.5 (27.4) | 0.002 |
| HDL-C, mg/dL^*^ | 53.4 (11.6) | | 52.7 (12.2) | 53.3 (11.3) | 53.7 (11.4) | 54.3 (11.3) | <0.001 |
| Triglycerides, mg/dL^†^ | 109.0 (82.0-150.0) | | 111.0 (81.0-156.0) | 111.0 (84.0-151.0) | 107.0 (81.0-147.0) | 107.0 (82.0-143.0) | 0.006 |
| Total bilirubin, mg/dL | 1.18 (0.48) | | 0.73 (0.17) | 1.04 (0.15) | 1.32 (0.17) | 1.90 (0.46) | <0.001 |
| Direct bilirubin, mg/dL | 0.44 (0.21) | | 0.30 (0.13) | 0.39 (0.13) | 0.48 (0.16) | 0.69 (0.22) | <0.001 |
| Albumin, g/dL | 4.43 (0.19) | | 4.40 (0.19) | 4.42 (0.19) | 4.45 (0.19) | 4.46 (0.20) | <0.001 |
| ALT, IU/L^†^ | 20.0 (16.0-25.0) | | 20.0 (16.0-25.0) | 21.0 (17.0-26.0) | 20.0 (16.0-25.0) | 20.0 (16.0-25.0) | 0.72 |
| AST, IU/L^†^ | 21.0 (19.0-24.0) | | 21.0 (19.0-24.0) | 21.0 (19.0-24.0) | 21.0 (19.0-24.0) | 21.0 (19.0-24.0) | 0.22 |
| GGT, IU/L^†^ | 20.0 (15.0-27.0) | | 20.0 (15.0-28.0) | 20.0 (15.0-27.0) | 20.0 (15.0-26.0) | 19.0 (15.0-27.0) | 0.01 |
| ALP, IU/L† | 54.0 (47.0-63.0) | | 55.0 (48.0-64.0) | 54.0 (47.0-63.0) | 54.0 (46.0-62.0) | 54.0 (46.0-62.0) | <0.001 |
| hsCRP, mg/L^†^ | 0.40 (0.20-0.80) | | 0.50 (0.20-1.00) | 0.40 (0.20-0.80) | 0.40 (0.20-0.70) | 0.30 (0.20-0.70) | <0.001 |
| Insulin, μU/dL | 6.33 (5.10-8.20) | | 6.73 (5.31-8.85) | 6.40 (5.11-8.38) | 6.12 (5.03-7.77) | 6.01 (4.86-7.64) | <0.001 |
| HOMA2-IR^†^ | 0.82 (0.66-1.07) | | 0.88 (0.69-1.14) | 0.83 (0.66-1.09) | 0.79 (0.65-1.02) | 0.78 (0.63-1.00) | <0.001 |

Data are ^*^means (standard deviation), ^†^medians (interquartile range), or percentages.

Abbreviations: ALT, alanine aminotransferase; AST, aspartate aminotransferase; BMI, body mass index; BP, blood pressure; GGT, gamma-glutamyltranspeptidase; HDL-C, high-density lipoprotein-cholesterol; hsCRP, high sensitivity C-reactive protein; HOMA-IR, homeostasis model assessment of insulin resistance; LDL-C: low-density lipoprotein-cholesterol.

^‡^ ≥20 g of ethanol per day.

^§^ ≥1 time/week.
