# Supplementary material for: A Cohort Study of Serum Bilirubin Levels and Incident Non-Alcoholic Fatty Liver Disease in Middle Aged Korean Workers
Source: PLoS One. 2012 May 15;7(5):e37241. doi: 10.1371/journal.pone.0037241 (PMC3352875; doi:10.1371/journal.pone.0037241)
Supplement: Table S3 — Baseline characteristics of study participants by quartile of serum direct bilirubin (N = 8,871). (DOC) [file pone.0037241.s003.doc]

**Table S3. Baseline characteristics of study participants by quartile of serum direct bilirubin (N = 8,871).**

|  | Overall | Direct bilirubin | | | | | *P* value for trend |
| --- | --- | --- | --- | --- | --- | --- | --- |
|  |  | Quartile 1 | | Quartile 2 | Quartile 3 | Quartile 4 |  |
| Number | 8871 | | 3399 | 1952 | 1404 | 2116 |  |
| Direct bilirubin, mg/dL^*^ | 0.44 (0.21) | | 0.25 (0.07) | 0.4 (0) | 0.5 (0) | 0.73 (0.17) |  |
| Range | 0 – 1.7 | | 0 – | 0.4 – | 0.5 – | 0.6 – 1.8 |  |
| Age, years^*^ | 37.0 (4.9) | | 37.1 (5.1) | 37.1 (4.9) | 36.8 (4.8) | 36.8 (4.8) | 0.005 |
| BMI, kg/m^2*^ | 23.3 (2.5) | | 23.6 (2.4) | 23.3 (2.5) | 23.1 (2.5) | 22.7 (2.4) | <0.001 |
| Current smoker, % | 46.6 | | 50.7 | 47.1 | 45.7 | 40.3 | <0.001 |
| Alcohol intake, %^‡^ | 40.2 | | 39.9 | 39.2 | 44.2 | 38.8 | 0.899 |
| Regular exercise, %^§^ | 51.3 | | 50.2 | 49.8 | 53.1 | 53.3 | 0.010 |
| Hypertension, % | 14.3 | | 14.1 | 15.0 | 14.9 | 13.6 | 0.718 |
| Metabolic syndrome, % | 6.8 | | 9.1 | 7.1 | 5.1 | 3.9 | <0.001 |
| Diabetes mellitus, % | 0.7 | | 0.7 | 0.6 | 0.7 | 0.9 | 0.512 |
| Cardiovascular disease, % | 0.2 | | 0.2 | 0.1 | 0.1 | 0.1 | 0.767 |
| Malignancy, % | 0.2 | | 0.2 | 0.2 | 0.2 | 0.3 | 0.628 |
| Lipid lowering agent, % | 0.5 | | 0.7 | 0.6 | 0.4 | 0.4 | 0.116 |
| Hemoglobin, g/dL | 15.1 (0.9) | | 15.0 (0.9) | 15.1 (0.8) | 15.2 (0.9) | 15.2 (0.9) | <0.001 |
| Leukocyte, x10^3^/μL | 5.9 (1.4) | | 6.1 (1.5) | 5.9 (1.4) | 5.8 (1.4) | 5.6 (1.3) | <0.001 |
| Systolic BP, mmHg^*^ | 115.0 (12.6) | | 115.0 (12.4) | 115.1 (12.6) | 115.0 (12.6) | 114.8 (12.9) | 0.610 |
| Diastolic BP, mmHg^*^ | 74.6 (10.0) | | 74.5 (9.9) | 74.9 (10.0) | 74.5 (10.3) | 74.4 (10.0) | 0.541 |
| Glucose, mg/dL^*^ | 90.8 (11.8) | | 92.3 (11.3) | 91.0 (11.4) | 90.6 (10.9) | 88.4 (13.1) | <0.001 |
| Uric acid, mg/dL^*^ | 5.88 (1.09) | | 5.88 (1.10) | 5.91 (1.09) | 5.90 (1.09) | 5.86 (1.09) | 0.532 |
| Total cholesterol, mg/dL^*^ | 197.7 (33.6) | | 205.0 (34.4) | 198.8 (33.1) | 193.7 (31.6) | 187.5 (30.9) | <0.001 |
| LDL-C, mg/dL^*^ | 117.4 (28.4) | | 122.0 (28.5) | 118.5 (28.1) | 115.0 (27.9) | 110.5 (27.4) | <0.001 |
| HDL-C, mg/dL^*^ | 53.6 (11.7) | | 51.2 (11.0) | 53.4 (11.7) | 54.4 (11.2) | 56.9 (12.4) | <0.001 |
| Triglycerides, mg/dL^†^ | 116.0 (86.0-161.0) | | 129.0 (94.0-179.0) | 119.0 (87.0-163.0) | 109.0 (82.0-152.0) | 100.0 (77.0-134.0) | <0.001 |
| Total bilirubin, mg/dL | 1.17 (0.47) | | 0.81 (0.20) | 1.08 (0.18) | 1.28 (0.21) | 1.77 (0.49) | <0.001 |
| Indirect bilirubin, mg/dL | 0.73 (0.31) | | 0.56 (0.18) | 0.68 (0.18) | 0.78 (0.21) | 1.04 (0.37) | <0.001 |
| Albumin, g/dL | 4.42 (0.20) | | 4.43 (0.20) | 4.42 (0.19) | 4.43 (0.20) | 4.42 (0.20) | 0.132 |
| ALT, IU/L^†^ | 22.0 (17.0-30.0) | | 24.0 (18.0-32.0) | 23.0 (18.0-31.0) | 22.0 (17.0-29.0) | 21.0 (17.0-28.0) | <0.001 |
| AST, IU/L^†^ | 23.0 (20.0-26.0) | | 23.0 (20.0-27.0) | 23.0 (20.0-27.0) | 22.0 (19.0-26.0) | 22.0 (19.0-26.0) | 0.001 |
| GGT, IU/L^†^ | 23.0 (16.0-34.0) | | 24.0 (17.0-37.0) | 23.0 (17.0-34.0) | 22.0 (16.0-34.0) | 21.0 (15.0-30.0) | <0.001 |
| ALP, IU/L† | 55.0 (47.0-63.0) | | 55.0 (48.0-64.0) | 55.0 (47.0-64.0) | 54.0 (47.0-62.0) | 54.0 (46.0-62.0) | <0.001 |
| hsCRP, mg/L^†^ | 0.40 (0.20-0.90) | | 0.50 (0.30-1.00) | 0.40 (0.20-0.90) | 0.40 (0.20-0.80) | 0.30 (0.20-0.70) | <0.001 |
| Insulin, µU/dL† | 6.56 (5.22-8.67) | | 7.13 (5.58-9.38) | 6.58 (5.31-8.59) | 6.24 (5.01-8.07) | 6.05 (4.93-7.79) | <0.001 |
| HOMA2-IR† | 0.86 (0.68-1.13) | | 0.93 (0.73-1.21) | 0.86 (0.69-1.12) | 0.81 (0.65-1.05) | 0.78 (0.63-1.00) | <0.001 |

Table S3 includes participants with liver conditions and risk factors for liver disease at baseline (except NAFLD). See text for details.

Data are ^*^means (standard deviation), ^†^medians (interquartile range), or percentages.

Abbreviations: ALT, alanine aminotransferase; AST, aspartate aminotransferase; BMI, body mass index; BP, blood pressure; GGT, gamma-glutamyltranspeptidase; ALP, alkaline phosphatase; HDL-C, high-density lipoprotein-cholesterol; hsCRP, high sensitivity C-reactive protein; HOMA-IR, homeostasis model assessment of insulin resistance; LDL-C: low-density lipoprotein-cholesterol.

^‡^ ≥20 g of ethanol per day.

^§^ ≥1 time/week.
