# Supplementary material for: A Cohort Study of Serum Bilirubin Levels and Incident Non-Alcoholic Fatty Liver Disease in Middle Aged Korean Workers
Source: PLoS One. 2012 May 15;7(5):e37241. doi: 10.1371/journal.pone.0037241 (PMC3352875; doi:10.1371/journal.pone.0037241)
Supplement: Table S4 — Hazard ratios (95% confidence intervals) for incident non-alcoholic fatty liver disease by serum bilirubin quartiles (N = 8,871). (DOC) [file pone.0037241.s004.doc]

**Table S4. Hazard ratios (95% confidence intervals) for incident non-alcoholic fatty liver disease by serum bilirubin quartiles (N = 8,871).**

|  | Person-years | No. of incident  Cases | Age-adjusted HR  (95% CI) | Multivariate HR^*^ (95% CI) | |
| --- | --- | --- | --- | --- | --- |
|  |  |  |  | Model 1 | Model 2 |
| Direct bilirubin (mg/dL) |  |  |  |  |  |
| 0 – | 15267.1 | 1395 | 1.00 (reference) | 1.00 (reference) | 1.00 (reference) |
| 0.4 – | 8893.4 | 713 | 0.87 (0.79-0.95) | 0.91 (0.83-0.99) | 0.96 (0.88-1.06) |
| 0.5 – | 6636.4 | 464 | 0.76 (0.68-0.84) | 0.85 (0.77-0.95) | 0.93 (0.84-1.04) |
| 0.6 – 1.8 | 10423.4 | 596 | 0.61 (0.56-0.67) | 0.76 (0.69-0.84) | 0.87 (0.79-0.96) |
| P for trend |  |  | <0.001 | <0.001 | 0.007 |
| Indirect bilirubin (mg/dL) |  |  |  |  |  |
| 0 – | 11243.9 | 926 | 1.00 (reference) | 1.00 (reference) | 1.00 (reference) |
| 0.6 – | 13482.5 | 1083 | 0.97 (0.89-1.06) | 0.95 (0.87-1.04) | 0.97 (0.89-1.06) |
| 0.7 – | 8527.0 | 616 | 0.87 (0.79-0.97) | 0.91 (0.82-1.01) | 0.93 (0.84-1.03) |
| 0.9 – 3.5 | 7966.8 | 543 | 0.82 (0.74-0.92) | 0.89 (0. 80-0.99) | 0.92 (0.83-1.03) |
| P for trend |  |  | <0.001 | 0.018 | 0.094 |
| Total bilirubin (mg/dL) |  |  |  |  |  |
| 0.3 – | 14210.6 | 1225 | 1.00 (reference) | 1.00 (reference) | 1.00 (reference) |
| 1.0 – | 8529.3 | 724 | 0.98 (0.89-1.08) | 0.99 (0.90-1.08) | 1.02 (0.93-1.12) |
| 1.2 – | 9423.3 | 632 | 0.77 (0.70-0.84) | 0.85 (0.77-0.94) | 0.90 (0.82-1.00) |
| 1.5 – 4.7 | 9056.9 | 587 | 0.75 (0.68-0.82) | 0.87 (0.79-0.96) | 0.94 (0.85-1.04) |
| P for trend |  |  | <0.001 | <0.001 | 0.058 |

Table S4 includes participants with liver conditions and risk factors for liver disease at baseline (except NAFLD). See text for details.

Model 1: Adjusted for age, BMI, current smoking, alcohol intake, exercise, diabetes mellitus, history of cardiovascular disease, history of malignancy, hepatitis B (positive for hepatitis B surface antigen), hepatitis C (positive for anti-HCV antibody), abnormal finding of liver ultrasound finding (ultrasonographic findings of chronic liver disease, liver cirrhosis, cholelithiasis, or abnormal dilatation of the biliary tree), AST and ALT

Model 2: Further adjusted for HDL cholesterol, triglycerides, glucose, insulin, and uric acid.

Abbreviations: BMI, body mass index; CI, confidence intervals; HDL-C, high-density lipoprotein-cholesterol; HR, hazard ratios.
